# Evaluation of the Avahan HIV prevention initiative in India

**DOI:** 10.1186/1471-2458-11-S6-I1

**Published:** 2011-12-29

**Authors:** Lalit Dandona, Eric G Benotsch

**Affiliations:** 1 Public Health Foundation of India, ISID Campus, 4 Institutional Area, Vasant Kunj, New Delhi 110070, India; 2 Institute for Health Metrics and Evaluation, University of Washington, Seattle, WA, USA; 3 Department of Psychology, Virgina Commonwealth University, Richmond, VA, USA

## 

The Avahan HIV prevention initiative is a major effort funded by the Bill and Melinda Gates Foundation that aims to reduce HIV transmission in the general population by focusing HIV prevention efforts on female sex workers and their clients, men who have sex with men, truck drivers and injecting drug users who are at high risk of getting HIV [1]. Avahan has been implemented in six states of India since 2003 that have had a high HIV burden – four large states in southern India (Andhra Pradesh, Karnataka, Maharashtra, and Tamil Nadu) and two small states in northeast India (Manipur and Nagaland) through 134 local non-governmental organizations [2]. It is important to note that Avahan, while unique and substantial, complements the much larger national HIV prevention effort in India.

Avahan has had an extensive evaluation design [2], some findings from which have been published previously in a supplement in *AIDS* in late 2008 and in a supplement in *Sexually Transmitted Infections* in early 2010. This supplement of *BMC Public Health* has papers reporting the next set of findings from studies associated with the evaluation of Avahan. While interpreting these findings it would be useful to remember that baseline data prior to the start of Avahan implementation are not available and there were no control groups for comparison [3].

The first three papers in this supplement describe the changes over time in the coverage of female sex workers by HIV prevention programmes in the states of Andhra Pradesh, Maharashtra and Tamil Nadu, their reported safe sex practices and the prevalence of HIV and other sexually transmitted infections among them [4-6]. The exposure to prevention programmes is reported to have increased in all states with consistent condom use with clients increasing from 64-76% to 84-95% from 2006 to 2009 in two cross-sectional surveys of 3200 female sex workers in each round in Andhra Pradesh, 2500 in Maharashtra and 2000 in Tamil Nadu. During this period, the HIV prevalence in the two cross-sectional samples of female sex workers is reported to have decreased from 17.7% to 13.2% in Andhra Pradesh and increased slightly from 25.8% to 27.5% in Maharashtra. Though both of these changes were statistically significant at the 95% confidence level, the difference in sample composition in the two cross-sectional surveys in 2006 and 2009 and other potential confounders make it difficult for these changes to be directly associated with Avahan. HIV prevalence among the female sex workers surveyed in Tamil Nadu remained constant at 6.1% over this period. Reactive syphilis serology, indicator of ever having had syphilis, declined significantly in all three states among surveyed female sex workers from 2006 to 2009, which is encouraging.

Analysis of data collected in 2006-2007 on about a 1000 female sex workers from four districts in Karnataka reports that half of the female sex workers had a husband or a co-habitating partner and a quarter had non-paying sex partner [7]. Consistent condom use was reported low at 23% with the former and 40% with the latter, and longer duration of partnership was associated with lower condom use. Analysis of three of these districts indicated that increased contact with HIV prevention staff and demonstration of condom use had a positive dose-response relation between condom use with paying clients but not with husbands or regular non-paying partners [8]. An analysis of risk perception among 5400 mobile female sex workers from Andhra Pradesh, Karnataka, Maharashtra and Tamil Nadu in 2007-2008 reports that only 40% of the sex workers considered themselves to be at high risk of HIV [9]. The current risk perception was related to previous condom use – those who had previously used condoms consistently with occasional paying clients perceived themselves to be at high risk and those who had used condoms inconsistently with non-paying partners perceived themselves to be at low risk. Importantly, the cross-sectional surveys in Andhra Pradesh, Maharashtra and Tamil Nadu mentioned above report continuing low consistent use of condoms with regular partners at 9-22% in 2009 [4-6]. This crucial issue needs to be addressed more effectively in HIV prevention programmes in India.

The next paper in this supplement reports 11% condom breakage with clients in the last month in a survey of 1900 female sex workers in 2008-2009 from four districts in Karnataka [10]. Younger, marginalized and those sex workers who had not had exposure to condom use demonstration had higher likelihood of experiencing condom breakage, indicating the need for particular attention to these sub-groups of sex workers for more effective HIV prevention. Analysis of data from two cross-sectional surveys of about 2000 trucker drivers on major national highways in each round reports an increase in exposure of truck drivers to HIV prevention programmes from 2007 to 2010 [11]. Consistent condom use was reported to have increased from 67% to 73% with paid female sex partners and from 18% to 37% with non-paid partners, and those with exposure to prevention programmes were more likely to report consistent condom use. Another paper reports that in a survey of 2700 male clients of female sex workers in six districts of Karnataka in 2008, the prevalence of HIV was 5.6% and of HSV-2 was 28.4%. Duration of paying for sex was associated with increased risk for these infections and infection risks differed by the type of commercial sex venue [12]. Data from about 1000 households each in one district in southern Andhra Pradesh and one district in northern Uttar Pradesh report that migrant men are likely to initiate high-risk sex at place of origin before migrating, and continue this at the place of migration and on return to the place of origin [13].

In the two northeastern states of Manipur and Nagaland, where the HIV epidemic is driven primarily by injecting drug use, data on two cross-sectional samples of about 1600 injecting drug users each in 2007 and 2009 suggest reduced frequency of needle sharing and increased condom use by injecting drug users with casual sex partners [14]. Exposure to intervention programmes was associated with lower needle sharing.

Over 331,000 female sex workers, 89,000 men who have sex with men and transgenders, and 10,000 injecting drug users visited sexually transmitted infection clinics through Avahan between 2005 and 2009 [15]. The rate of diagnosis of sexually transmitted infections decreased substantially over this period. The high coverage of over 430,000 persons at risk of HIV is a good achievement. Another paper in this supplement analyses the cost of HIV prevention services in Bangalore and Mumbai cities and reports that the unit cost was higher for harder to reach men who sell sex to men and transgenders than for female sex workers [16].

A paper on population-based surveys from three districts of Karnataka done during 2006-2008 reports heterogeneity in the stage of the HIV epidemic in different parts of the state [17]. Two cross-sectional population-based surveys in a district of Karnataka in 2003 and 2009 report that in the second survey a higher proportion of respondents, especially women, endorsed the view that access to condoms and sex education increases promiscuity [18]. Such attitudes, if widespread, would make HIV prevention more difficult.

Avahan should be commended for making learning from evaluation a crucial part of its effort [2]. On the other hand, there have also been some limitations of the evaluation design – no baseline before intervention, no control groups, and predominant focus on assessing the impact in high-risk groups even though the final goal of Avahan was to reduce transmission of HIV in the general population. A recent paper in the *Lancet* that used national HIV sentinel surveillance data and modeling techniques to relate Avahan intervention intensity with trends in HIV prevalence over time while adjusting for the various potential confounders found that Avahan intensity was significantly associated with lower HIV prevalence in three south Indian states and it prevented about 100,000 HIV infections in its first phase from 2003-2008 [19]. This estimated beneficial effect of Avahan at the population level is encouraging. However, it is important to understand through more detailed studies the processes through which this effect would have manifested. Some such insights are provided by the papers in this supplement and more will perhaps follow.

On a broader note, an analysis of evaluations of population health interventions in India has reported that the evaluations are generally poor in design and analytical approaches [20]. The evaluation effort of Avahan offers useful insights into how evaluation of large-scale health programmes in India could be strengthened.
